# Phylogenetic representativeness: a new method for evaluating taxon sampling in evolutionary studies

**DOI:** 10.1186/1471-2105-11-209

**Published:** 2010-04-27

**Authors:** Federico Plazzi, Ronald R Ferrucci, Marco Passamonti

**Affiliations:** 1 Department of "Biologia Evoluzionistica Sperimentale", University of Bologna, Via Selmi, 3 - 40126 Bologna, Italy; 2 Department of Biology and Evolution, University of Ferrara, Via Borsari, 46 - 44100 Ferrara, Italy

## Abstract

**Background:**

Taxon sampling is a major concern in phylogenetic studies. Incomplete, biased, or improper taxon sampling can lead to misleading results in reconstructing evolutionary relationships. Several theoretical methods are available to optimize taxon choice in phylogenetic analyses. However, most involve some knowledge about the genetic relationships of the group of interest (i.e., the ingroup), or even a well-established phylogeny itself; these data are not always available in general phylogenetic applications.

**Results:**

We propose a new method to assess taxon sampling developing Clarke and Warwick statistics. This method aims to measure the "phylogenetic representativeness" of a given sample or set of samples and it is based entirely on the pre-existing available taxonomy of the ingroup, which is commonly known to investigators. Moreover, our method also accounts for instability and discordance in taxonomies. A Python-based script suite, called PhyRe, has been developed to implement all analyses we describe in this paper.

**Conclusions:**

We show that this method is sensitive and allows direct discrimination between representative and unrepresentative samples. It is also informative about the addition of taxa to improve taxonomic coverage of the ingroup. Provided that the investigators' expertise is mandatory in this field, phylogenetic representativeness makes up an objective touchstone in planning phylogenetic studies.

## Background

The study of phylogenetics has a long tradition in evolutionary biology and countless statistical, mathematical, and bioinformatic approaches have been developed to deal with the increasing amount of available data. The different statistical and computational methods reflect different ways of thinking about the phylogeny itself, but the issue of "how to treat data" has often overshadowed another question, i.e., "where to collect data from?". We are not talking about the various types of phylogenetic information, such as molecular or morphological characters, but rather we refer to which samples should be analyzed.

In phylogenetic studies, investigators generally analyze subsets of species. For example, a few species are chosen to represent a family or another high-level taxon, or a few individuals to represent a low-level taxon, such as a genus or a section. As a general practice, choices are driven by expertise and knowledge about the group; key species and taxa of interest are determined and, possibly, sampled. For example, if a biologist is choosing a group of species to represent a given class, species from many different orders and families will be included. We term the degree to which this occurs the "phylogenetic representativeness" of a given sample.

This issue is rarely formally addressed and generally treated in a rather subjective way; nevertheless, this is one of the most frequent ways incongruent phylogenetic results are accounted for. It is sufficient to browse an evolutionary biology journal to see how often incorrect or biased taxon sampling is hypothesized to be the cause [e.g., [1-6]]. We therefore aim to set up a rigorous taxon sampling method, which can be used alongside expertise-driven choices. Many theoretical approaches have been proposed to drive taxon sampling: see [[7]; and reference therein] for a keystone review.

The concept of "taxonomic distinctness" was developed in the early 1990s among conservation biologists [8,9], who needed to measure biodiversity within a given site or sample so to assess further actions and researches. Basic measures of biodiversity take into account species richness and relative abundance [10-13]. However, it is clear from a conservationist point of view that not all species should be weighted the same. The presence and relative abundance of a species cannot capture all information on the variation of a given sample, and therefore a taxonomic component must also be considered in evaluating the biodiversity of a given site. This allows more realistic specification of the importance of a species in a given assemblage.

Similarly, resources for conservation biology are limited, and therefore it is important to focus on key species and ecosystems according to a formal criterion. For this purpose, several methods have recently been proposed [14-17]. Despite recent progresses in sequencing techniques, it is still worth following a criterion of "maximizing representativeness" to best concentrate on key taxa [e.g., [17]]. Nevertheless, this typically requires a well established phylogeny, or at least a genetic distance matrix, as a benchmark. These data are indeed generally available for model species or taxa with key ecological roles, but they are often unavailable in standard phylogenetic analyses. Typically, if we want to investigate a phylogeny, it has either never been resolved before, or it has not been completely assessed at the moment we start the analysis. Further, if a reliable and widely accepted phylogenetic hypothesis were available for the studied group, we probably would not even try to attempt to formulate one at all. This means that, while the above-mentioned methods may be useful in the case of well-characterized groups, an approach using taxonomic distinctness is more powerful in general phylogenetic practice.

Our basic idea is that estimating the phylogenetic representativeness of a given sample is not conceptually different from estimating its taxonomic distinctness. A certain degree of taxonomic distinctness is required for individual samples chosen for phylogenetic analyses; again, investigators attempt to spread sampling as widely as possible over the group on which they are focusing in order to maximize the representativeness of their study. A computable measure of taxonomic distinctness is required to describe this sampling breadth.

In this article we propose a measure of phylogenetic representativeness, and we provide the software to implement it. The procedure has the great advantage of requiring only limited taxonomical knowledge, as is typically available in new phylogenetic works.

## Results

### Algorithm

Clarke and Warwick [18] suggest standardizing the step lengths in a taxonomic tree structure by setting the longest path (i.e., two species connected at the highest possible level of the tree) to an arbitrary number. Generally, this number is 100. Step lengths can be weighted all the same, making the standardized length measure to equal:

where *T *is the number of taxonomic levels considered in the tree and *n = 1*, *2*, ..., *N*, where *N *is the number of steps connecting a pair of taxa (see Methods). All taxa in the tree belong by definition to the same uppermost taxon. Therefore, two taxa can be connected by a maximum of *2(T - 1) *steps.

However, it is also possible to set step lengths proportionally to the loss of biodiversity between two consecutive hierarchical levels, i.e., the decrease in the number of taxa contained in each one, as measured on the master list. Branch lengths are then computed as follows: we indicate *S*_(*t*) _as the number of taxa of rank *t*, with *t *= *1*, *2*, ..., *T *from the lowest to the highest taxonomic level. Two cases are trivial: when *t = 1*, *S*_(*t*) _equals to *S *(the number of Operational Taxonomic Units - OTUs - in the master taxonomic tree); when *t = T*, *S*_(*t*) _equals to *1 *(all taxa belong to the uppermost level). The loss of biodiversity from level *t *to level *t + 1 *is:

The step length from level *t + 1 *to level *t *is the same as from level *t *to level *t + 1*. Therefore, path lengths are then obtained as:

where *l*_*t *_is the path length from level *t *to level *t + 1 *and *l*_t* _is the reverse path length.

Clarke and Warwick [18] found the method of weighting step lengths to have little effect on final results. However, we find that standardizing path lengths improves the method in that it also complements subjectivity in taxonomies; rankings are often unrelated even across closely-related groups. To us, this is the main reason for standardizing path lengths. Moreover, adding a level in a taxonomic tree does not lead to changes in the mean or standard deviation of taxonomic distance (AvTD or VarTD) if we adopt this strategy. In addition, the insertion of a redundant subdivision cannot alter the values of the indices [18]. All these analyses are carried out by our PhyRe script (Additional file 1).

Our method based on Clarke and Warwick's ecological indices has the main feature of being dependent only upon a known existing taxonomy. This leads to a key difficulty: taxonomic structures are largely subjective constructions. Nonetheless, we think that taxonomists' expertise has provided high stability to main biological classifications, at least for commonly-studied organisms, such as animals and plants. The degree of agreement which is now reached in those fields allows us to consider most systematics as stable. In our view, large-scale rearrangements are becoming more and more unlikely, so that this argument leads us to state that present taxonomies do constitute an affordable starting point for methods of phylogenetic representativeness assessment.

However, this is not sufficient to completely ensure the reliability of our method. Knowledge is growing in all fields of evolutionary biology, and the increase in data results in constant refinement of established classifications. In fact, even if large-scale changes are rare, taxonomies are frequently revised, updated, or improved. Therefore, we implemented an algorithm that allows for testing the stability of the chosen reference taxonomy.

Essentially, our procedure can be described in two phases. In the first one, the shuffling phase, master lists are shuffled, resulting in a large number of alternative master lists. In the second, the analysis phase, a phylogenetic representativeness analysis is carried out as described above across all simulated master lists rearrangements. The shuffling phase is composed of three moves, which are repeated and combined *ad libitum *(see Methods). These moves simulate the commonest operations taxonomists do when reviewing a classification. A large number of "reviewed" master lists is then produced, repeating each time the same numbers of moves. Finally, the shuffling phase ends with a set of master lists. Standard phylogenetic representativeness analyses are performed on each master list, and all statistics are computed for each list. In this way, a set of measurements is produced for each indicator. Therefore, it is possible to compute standard 95% (two-tailed) confidence intervals for each one. This analysis phase gives an idea of the funnel plot's oscillation width upon revision. PhyloSample and PhyloAnalysis (Additional file 1) are specific scripts dealing with the shuffling analysis: the former generates the new set of master list, whereas the latter performs PhyRe operations across them all.

All scripts are available online, and a Windows executable version of the main script is also present: the software can be downloaded from the MoZoo Lab web site at http://www.mozoolab.net/index.php/software-download.html.

### Testing

In order to evaluate the method, we analyze phylogenies of bivalves [19], carnivores [20], coleoids [21], and termites [22]. Our reference taxonomies are Millard [23] for mollusks, the Termites of the World list hosted at the University of Toronto http://www.utoronto.ca/forest/termite/speclist.htm: consulted on 03/23/2009 and reference therein), and the online Checklist of the Mammals of the World compiled by Robert B. Hole, Jr. (http://www.interaktv.com/MAMMALS/Mamtitl.html: consulted on 03/11/2009 and reference therein).

**Figure 1:**
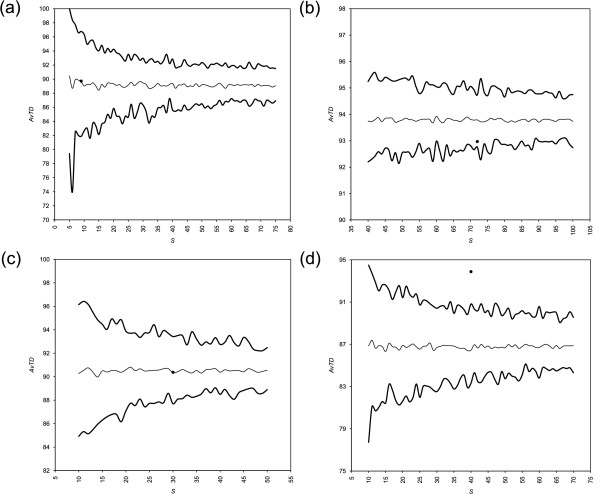
**Funnel plots of AvTD from four published data sets**. Funnel plots of Average Taxonomic Distinctness (AvTD) from (a) bivalves [19], (b) carnivores [20], (c) coleoids [21], and (d) termites [22] data sets are shown. Results are from 100 random replicates. Thick lines are the highest values found across all replicates of each dimension and the lower 95% confidence limit; the thin line is the mean across all replicates; experimental samples are shown by black dots.

**Figure 2:**
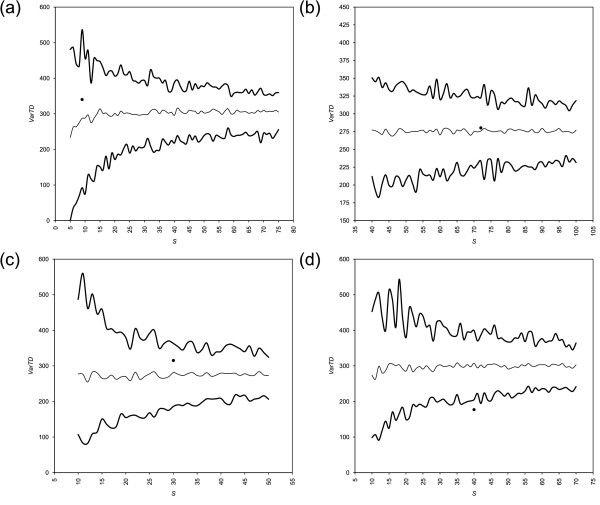
**Funnel plots of VarTD from four published data sets**. Funnel plots of Variation in Taxonomic Distinctness (VarTD) from (a) bivalves [19], (b) carnivores [20], (c) coleoids [21], and (d) termites [22] data sets are shown. Results are from 100 random replicates. Thick lines are the upper 95% confidence limit and the lowest values found across all replicates of each dimension; the thin line is the mean across all replicates; experimental samples are shown by black dots. The bias towards lower values for small sample is detectable in mean.

Results from AvTD and VarTD are shown in Figures 1 and 2, respectively. Funnel plot are based arbitrarily on 100 random samplings from the master list for each sample size. Table 1 summarizes these results, showing also results from *I*_*E*_.

**Table 1:** Phylogenetic Representativeness analyses from four published works.

Group	Reference	Dimension	AvTD	VarTD	I_E_
Bivalves	[19]	9	89.7181	340.1874	0.0609
Carnivores	[20]	72	92.9688	280.2311	0.1203
Coleoids	[21]	30	90.3758	315.3069	0.1079
Termites	[22]	40	93.8788	177.1053	0.1631

Dimension, number of taxa; AvTD, Average Taxonomic Distinctness; VarTD, Variation in Taxonomic Distinctness; *I*_*E*_, von Euler's [44] Index of Imbalance.

**Figure 3:**
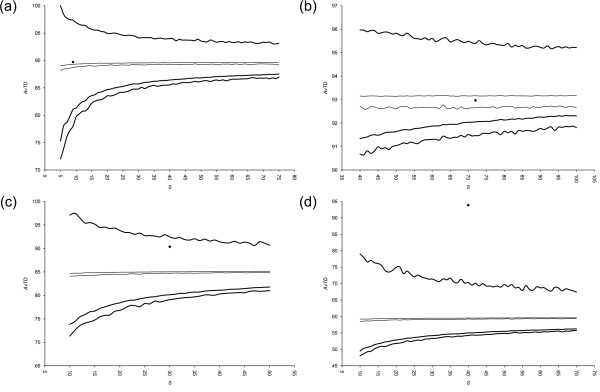
**Funnel plots of AvTD from shuffling analyses**. Funnel plots of Average Taxonomic Distinctness (AvTD) upon master lists' shuffling from (a) bivalves [19], (b) carnivores [20], (c) coleoids [21], and (d) termites [22] data sets are shown. Results are from 1,000 shuffled master lists and 100 random replicates. Thick lines are the highest values found across all replicates and the lower 95% confidence limit (2.5% and 97.5% confidence limits); thin lines represent the mean across all replicates (2.5% and 97.5% confidence limits); experimental samples are shown by black dots. Shuffling tuning as in Table 2.

**Figure 4:**
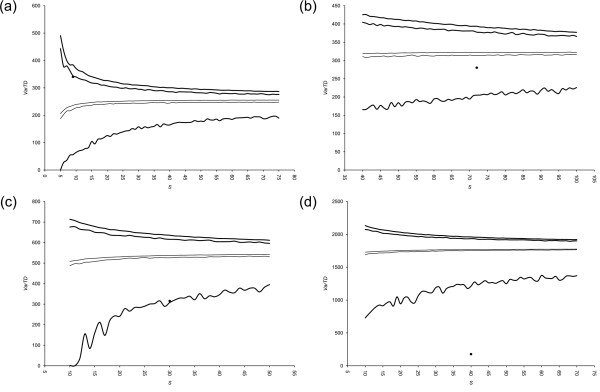
**Funnel plots of VarTD from shuffling analyses**. Funnel plots of Variation in Taxonomic Distinctness (VarTD) upon master lists' shuffling from (a) bivalves [19], (b) carnivores [20], (c) coleoids [21], and (d) termites [22] data sets are shown. Results are from 1,000 shuffled master lists and 100 random replicates. Thick lines are the upper 95% confidence limit (2.5% and 97.5% confidence limits) and the lowest values found across all replicates; thin lines represent the mean across all replicates (2.5% and 97.5% confidence limits); experimental samples are shown by black dots. Shuffling tuning as in Table 2.

To assess the stability of our taxonomies by performing shuffling analyses on them, we fixed the amount of "moves" to be executed according to our knowledge of each master list (see Discussion for details; Table 2); 1,000 new "reviewed" datasets were generated and then 100 replicates were again extracted from each master list for each sample size. Funnel plots for AvTD and VarTD are shown in Figures 3 and 4, respectively.

**Table 2:** Shuffling moves performed on each master list.

Group	Size	Level	Splits	Merges	Transfers
Bivalves	3404	Family	15	10	40
Carnivores	271	subfamily	2	1	2
Coleoids	220	Family	2	1	2
Termites	2760	species	0	0	15

Each set of splits, merges, and transfers was repeated independently 1,000 times on the relative master list. Moves were applied to the specified taxonomic level. Master list's size is reported to inform about the entity of the "reviewing" shuffle. Size in Operational Taxonomic Units (OTUs) of the global taxonomic tree.

We conducted additional analyses on the dataset of bivalves with real and simulated data (Additional file 2). Data from bivalve phylogenies obtained in our laboratory at different times from different samples have been tested along with imaginary samples of different known representativeness. We use the letter R to denote real data sets analyzed in our laboratory. Datasets from R1 to R4 are increasingly representative. In R1, the subclass of Protobranchia is represented by just one genus, and the subclass of Anomalodesmata is completely missing. In R2, we add one more genus to Protobranchia (*Solemya*) and one genus to Anomalodesmata (*Thracia*). In R3, the sample is expanded with several Genera from Unionidae (*Anodonta*, *Hyriopsis*), Heterodonta (*Gemma*, *Mactra*), Protobranchia (*Nuculana*; but see [24,25]), and more Anomalodesmata (*Pandora*, *Cuspidaria*). While all high-level taxa were already represented in R2, R3 is thus wider and more balanced in terms of sampling. R4 is identical to R3 with the exception of genus *Cerastoderma*, which was excluded due to technical problems.

Simulated data sets are indicated by the letter S. S1 is an "ideal" data set: all subclasses are represented with 4 species and 4 families, although the number of represented orders is different across the subclasses. S2 is biased towards less biodiversity-rich subclasses: it comprehends 6 anomalodesmatans, 6 palaeoheterodonts, and 7 protobranchs, along with only 1 pteriomorphian and one heterodont. S3 is strongly biased towards heterodonts, with 17 genera. Pteriomorphians, palaeoheterodonts, and protobranchs are represented by one genus each, and there are no anomalodesmatans here. S4 is an "easy-to-get" sample, with the commonest and well-known genera (e.g., *Donax*, *Chamelea*, *Teredo*, *Mytilus*, *Ostrea*), and therefore it is composed only by pteriomorphians (7 genera) and heterodonts (11 genera).

**Figure 5:**
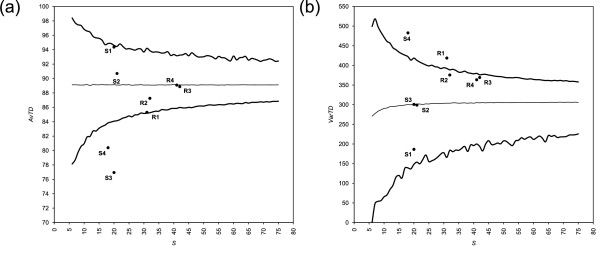
**AvTD and VarTD from bivalve data sets**. Phylogenetic Representativeness as measured by funnel plots of (a) Average Taxonomic Distinctness (AvTD) and (b) Variation in Taxonomic Distinctness (VarTD) from bivalves' master list [23]. Results are from 10,000 random replicates. Lines are as in Figure 1 and 2 for (a) and (b), respectively. Letter S denotes simulated data sets, whereas letter R denotes real ones. See text for explanation.

For this entire group of samples, from R1 to R4, and from S1 to S4, we conducted phylogenetic representativeness analyses to find out whether the method can describe samples following our expectations. Funnel plots were constructed on 10,000 replicates. Results are displayed in Figure 5 and Table 3.

**Table 3:** Phylogenetic representativeness across real and simulated bivalve data sets.

Sample	Group	Dimension	AvTD	VarTD	I_E_
*real*					
R1	without anomalodesmatans	31	85.3003	418.7537	0.2586
R2	+ *Solemya *and *Thracia*	32	87.2497	375.5878	0.2804
R3	increased (see text)	42	88.8653	369.2571	0.1806
R4	- *Cerastoderma*	41	89.0842	363.4391	0.1773
*simulated*					
S1	"ideal" (see text)	20	94.3673	186.2882	0.0476
S2	biased towards poor subclasses	21	90.6962	298.9607	0.1676
S3	biased towards heterodonts	20	76.9450	300.7505	0.7017
S4	"easy-to-get" (see text)	18	80.3913	482.7998	0.2419

Dimension, number of taxa; AvTD, Average Taxonomic Distinctness; VarTD, Variation in Taxonomic Distinctness; *I*_*E*_, von Euler's [44] Index of Imbalance.

### Implementation

**Figure 6:**
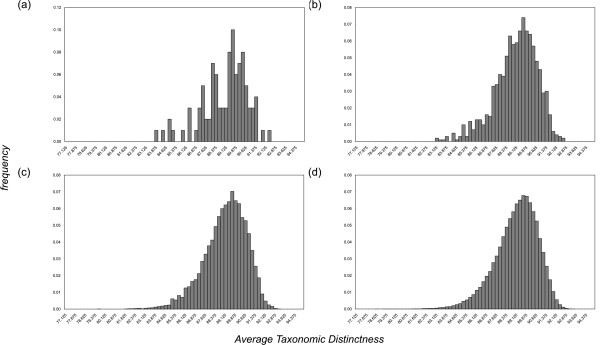
**Average Taxonomic Distinctness distribution**. Histograms show frequencies of Average Taxonomic Distinctness (AvTD) values among *k *= 100 (a), 1,000 (b), 10,000 (c), and 100,000 (d) random subsamples (*S *= 50) from bivalves' master list by Millard [23]. The distribution shows a skeweness towards the left side.

The distribution of AvTD from *k *random subsamples of size *S *is typically left-skewed ([26]; Figure 6). This is not an effect of a low *k*, as increasing the number of subsamples the shape of distribution does not change. We follow Azzalini [27] in describing the skeweness with a parameter λ. The further is λ (as absolute value) from unity, the more skewed is the distribution. Using the master list of bivalves and a dimension *S *of 50, we estimated an absolute value for λ which is very close to unity (~1.01, data not shown), confirming that the distribution only slightly differs from the normal one. However, this was done only for one sample, and distributions vary across different taxonomies and organisms. Similar considerations can be applied to VarTD.

We represent in our AvTD plots the lower 95% confidence limit (see Figures from 1 to 5). The maximum value obtained across all replicates for that dimension is also shown because it converges to the upper absolute limit as *k *increases. Conversely, in VarTD plots the upper 95% confidence limit and minimum observed value are shown, as lower values of variation are preferable (see Methods). PhyRe produces funnel plots showing results from a range of dimensions *S*. This helps in evaluating the global situation and is very useful for comparing homogeneous samples of different sizes.

For the shuffling analysis, similar funnel plots are produced. The main difference is that for AvTD the lower 95% confidence limit is not a line: here is shown the area which comprises 95% of values for each dimension across all shuffled master lists. The same applies for the AvTD and VarTD means, and the VarTD upper 95% confidence limit.

Output from PhyRe can easily be imported into a graph editing software like Microsoft Excel^®^.

## Discussion

"Taxon sampling" is not a new topic by itself and several strategies have been proposed from different standpoints. As mentioned above, several criteria have been appraised, especially when an established phylogeny is present. Long-branch subdivision [[28,29]; and reference therein], for example, has been proposed as one strategy; see Hillis [[7]; and reference therein] for more strategies. Much experimental interest has been focused also on outgroup sampling (see, e.g., [[30,31]; and reference therein], for empirical studies) and its effects. Finally, whether it is preferable to add more characters or more taxa is a vexing question; several authors highlight the importance of adding new taxa to analyses [e.g., [32,33]]. However, Rokas and Carroll [34] point out that an increase in taxon sampling does not have an improving effect *per se*. Nevertheless, they suggest several factors which may influence the accuracy of phylogenetic reconstructions, and among them the density of taxon sampling.

Rannala et al. [35] obtained more accurate phylogenetic reconstructions when they sampled 20 taxa out of 200, rather than when 200 taxa out of 200,000 were chosen for analyses, although in the latter case the taxon number was higher. This is rather intuitive, indeed, as taxon sampling is denser in the former case. Each taxon was sampled with the same probability ρ in a birth-death process (see [35] for further details). Interestingly, this is somewhat similar to our random subsampling process: the more dense is a sample, the more likely is it to be representative of its master list, despite the absolute number of included taxa.

However, our approach is very different, because it is completely *a priori*. The method can always be applied to any phylogeny, given the presence of a reference taxonomy and a master list of taxa. We find useful to start from the zero point of no phylogenetic information except for the available taxonomy. Evolutionary systematics does indeed capture some phylogenetic information, because all taxonomic categories should correspond to monophyletic clades. We employ this preliminary phylogenetic information to assess taxon sampling (but see below for further discussion on this point).

This method can be applied to every kind of analysis, from molecular to morphological ones. Furthermore, even extinct taxa can be included in a master list or in a sample: for example, the bivalve list from Millard [23] does report fossil taxa, and we left those taxa in our reference master list, as these are part of the biodiversity of the class. In fact, a good sample aims to capture the entire diversity of the group, thus including extinct forms. Therefore, we suggest that molecular samples should be better compared to complete master lists, which comprehend both living and fossil taxa (see Figure 5).

Moreover, evaluating phylogenetic representativeness as described here has the great advantage of being largely size-independent: this is well shown by funnel plots of AvTD and VarTD (Figures from 1 to 5). The mean is consistent across all dimensions *S *and it is very close to AvTD or VarTD values obtained from the whole master list (data not shown; see e.g., [26]). This fact, along with setting path lengths proportionally to biodiversity losses and rescaling their sum to 100, has a very useful and important effect: adding new taxa or new taxonomic levels does not change any parameter in the analysis. This means that more and more refined analyses can always be addressed and compared with coarser ones and with results from other data.

Most importantly, we checked the significance of both AvTD and VarTD results with one-tailed tests. The original test was two-tailed [26], and this is the greatest difference between the original test and our implementation for phylogenetic purposes. In the ecological context, these indices are used to assess environmental situations, to test for ecological stresses or pollution. In such a framework, the index must point out assemblages which are either very poor or very rich in terms of distinctness. The former will constitute signals of critically degraded habitats, whereas the latter will indicate a pristine and particularly healthy locality, and ecologists seek explanations for both results.

In our applications, we want our sample to be representative of the studied group, so that a sample significantly higher in taxonomic distinctness than a random one of the same size can be very useful; indeed, it would be even preferred. For this reason, we state that a one-tailed test is more appropriate for our purposes.

All case studies rely on samples with good phylogenetic representativeness. Nevertheless, one sample ([19]; Figure 1a and 2a) is relatively small to represent its master list; this is shown by quite large funnels at its size. On the other hand, one sample ([22]; Figure 1d and 2d) turned out to be strikingly representative of its groups: the AvTD is higher (and the VarTD lower) than the highest (lowest) found in 100 random subsamples. We recommend the former sample be taken with care for phylogenetic inferences (in fact, see [19] on the polyphyly of bivalves). Conversely, the latter sample is extremely more representative than the other three. Highly representative samples are readily individuated by AvTD and VarTD funnel plots (see Figure 1d and 2d) as dots above the highest AvTD and below the lowest VarTD found across all random replicates.

This is naturally influenced by the number of such subsamples: the more subsamples that are drawn, the more likely is to find the absolute maximum (minimum) possible value. If *k *is sufficiently high, the absolute maximum (minimum) possible value is found for any dimension *S*, and no sample can appear above (below) those lines (see Figure 5). Therefore, we suggest to draw an intermediate number of replicates (e.g., 100 or 1,000) to avoid this widening effect and identify more optimal phylogenetic samples.

Shuffling analysis assesses the complex issue of master list subjectivity and, as such, taxonomy itself. Master lists turn out to be substantially stable upon simulated revision, as shown in Figure 3 and 4. 95% confidence areas are indeed generally narrow and the position of experimental dots is never seriously challenged. We used 100 replicates from 1,000 master lists: this turned out to be sufficient to draw clear graphs, where borders are accurately traced.

An objective criterion to describe the amount of shuffling needed for this analysis is still lacking; however, each group of living beings has its own taxonomic history and its own open problems, therefore we think it can be very difficult to find an always-optimal criterion. An expertise-driven choice cannot be ruled out here. We suggest that, given the contingent conditions of a study, phylogeneticists choose the best degree of shuffling to describe their master list's stability. Some taxonomical situations are much more consolidated than others; in some cases higher-level taxa are well-established, whereas in others agreement has been reached on lower-level ones. A formal criterion, like moving 10% of species or merging 5% of genera, will necessarily lose this faceting and complexity.

Interestingly, the coleoid master list revealed itself to be the most sensitive to shuffling. The AvTD funnel plot places the sample of [21] exactly across the mean line, whereas it is close to the maximum line in the shuffling analysis (see Figure 1c and 3c). This means that AvTD is globally lowered upon shuffling on the coleoid master list. In fact, whereas mean AvTD on the original master list was close to 90 for all *S*, the 95% confidence interval on shuffled master lists is always slightly under 85. Conversely, VarTD is over the mean in standard PhyRe computations, whereas it is across the minimum line in shuffling analysis (see Figure 2c and 4c): VarTD mean changes from about 300 in the former case to around 500 in the latter one. The amount of shuffling we applied (see Table 2) is evidently heavy in this case. Therefore, upon a taxonomic review, we would recommend to reconsider this sample and to perform a new phylogenetic representativeness analyses.

Our method is also descriptive for comparing similar samples; this is a smart way to test the improvement of a phylogenetic study while adding one or more taxa to a given sample. It is clear from our R1-R4 example (see Figure 5) the importance of adding just two taxa to the initial sample. The improvement is well depicted by AvTD and VarTD funnel plots: whereas R1 is just across the AvTD lower 95% confidence limit of AvTD, R2 is well above; whereas R1 is outside the VarTD upper 95% confidence limit, R2 is inside it. While VarTD remains close to the confidence limit, R3 and R4 are nevertheless even more representative in terms of AvTD, as they lie precisely on the mean of 10,000 replicates. This reflects the increase of sampled taxa with respect to several under-represented groups.

S1, the "ideal" sample, turns out to have the highest AvTD (across the maximum line) and the lowest VarTD (next to the minimum line). In this case, we have 10,000 replicates; thus, the above considerations hold true and we do not expect our dot to be neither above nor below the funnel plot for AvTD or VarTD, respectively. Sample S2, biased towards less biodiversity-rich subclasses appears to be representative: it is well inside both funnel plots. Three subclasses out of five are well represented here; this sample is therefore rather informative. However, it is clearly less preferable than sample S1; whereas the former lies always across or next to the mean line, the latter is always close to the observed extreme values. Sample S3 seems reasonable in terms of VarTD, but the AvTD funnel plot identifies it as the worst of all. Nevertheless, sample S4 (with two substantially equally-represented subclasses) turned out to be even worse than S3 (almost just one subclass included): it is below the 95% confidence limit of AvTD and above the 95% confidence limit of VarTD.

**Figure 7:**
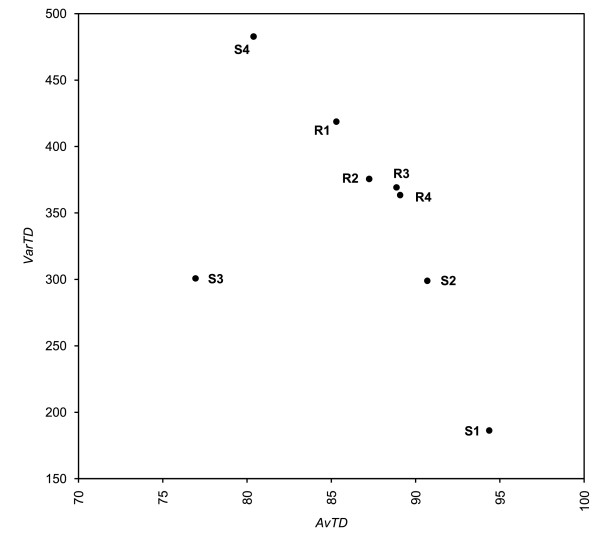
**AvTD-VarTD plot**. Variation in Taxonomic Distinctness (VarTD) plotted on Average Taxonomic Distinctness (AvTD) for real and simulated bivalve datasets (see Table 3 for further details on samples).

Thus, joint analysis of AvTD and VarTD provides discrimination between samples. An AvTD/VarTD plot shows that these measures are generally negatively correlated, even if some exceptions are possible: good samples have high AvTD and low VarTD values; the opposite is true for bad samples (Figure 7).

Along with the two main measures, *I*_*E *_can give an approximate idea of the shape of the tree. Values > 0.25 are often associated with biased samples (see Table 3), and thus we suggest this as a rule of thumb for directly discarding imbalanced ones. However, this cut-off value is only a rough guide in estimating phylogenetic representativeness: sample R2 has an *I*_*E *_of 0.2804 (greater than R1), but funnel plots identify it as a good bivalve sample.

## Conclusions

Phylogenetic representativeness analyses can be conducted at every taxonomic level, and including any taxonomic category. Moreover, inclusion or exclusion of taxonomic categories does not influence results across analyses ([18]; see above). Although we did not present it here, the index can also potentially take relative abundance data into account [see [36,37,26]]. Thus, it may be implemented for population-level analyses as well, depicting sampling coverage among different populations from a given section, species, or subspecies.

The main strength of phylogenetic representativeness approach lies in being an *a priori *strategy of taxon selection and sampling. Therefore, it cannot take into account several empirical and experimental problems, which are not guaranteed to be avoided. For example, long-branch attraction depends essentially upon a particularly quick rate of evolution in single taxa [38], which is only *a posteriori *identified. Moreover, topology alteration due to outgroup misspecification remains possible, as phylogenetic representativeness deals only with ingroup taxa.

Each particular study copes with specific difficulties strictly inherent to contingent conditions; for example, as a result of an unexpected selective pressure, one particular locus may turn out to be completely uninformative, even if the taxon sampling is perfectly adequate. Nevertheless, in R1-R4/S1-S4 examples (see above), our knowledge of bivalve evolution and systematics allows us to discriminate between suitable and non-suitable samples, and phylogenetic representativeness results matched perfectly with our expectations.

Moreover, being understood that expertise is always expected in planning taxon sampling, we strongly suggest to set phylogenetic representativeness alongside a formal criterion for profiling phylogenetic informativeness of characters [e.g., [39]]. Put in other words, phylogenetic representativeness is a guarantee of a good and wise taxonomic coverage of the ingroup, but evidently it is not guarantee of a good and robust phylogeny *per se*. For this reason, we would suggest it as a springboard for every phylogenetic study, from which subsequent analyses can proceed further towards an affordable evolutionary tree.

## Methods

### Average Taxonomic Distinctness (AvTD)

Mathematical aspects of this index are well explained in works by Clarke and Warwick [36,26,40]. However, it is useful to explain here the main points of their statistics.

AvTD is computed starting from a taxonomic tree. A taxonomic tree is merely the graphical representation of a Linnean classification, whereby OTUs are arranged hierarchically into different categories or taxa, with taxa being mutually exclusive. We use the general terms "OTUs" and "taxa" because a taxonomic tree does not necessarily include species at their tips, nor do all taxonomic trees take into account exactly the same levels of systematics.

**Figure 8:**
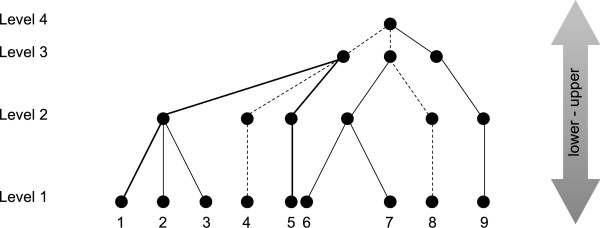
**A hypothetical taxonomic tree**. Nine Operational Taxonomic Units (OTUs) and four taxonomic levels are shown. For example, levels 1-4 could correspond to species, genera, families, and orders, respectively; in this case, species 1, 2, and 3 would belong to the same genus, species 1, 2, 3, and 4 to the same family, and so on. Taxonomic paths connecting taxa 1 and 5 (thick lines) and taxa 4 and 8 (dashed lines) are marked. See text for more details.

A simple taxonomic tree is depicted in Figure 8. Each leaf is an OTU and each node is a taxon; for example, OTUs may correspond to species and deeper nodes to genera, families, and orders as we climb up the tree. On a tree such as this, we can define a tree metric of taxonomic distance between any given pair of OTUs. A taxonomic tree is rooted (by definition); therefore, it is necessary to specify that our tree metric is unrooted (see [16]), i.e., the distance between two taxa is the shortest path on the tree that leads from one to another, and it is not required to climb up the tree from the first taxon to the root and then down to the second one, otherwise all pairs of OTUs would score the same distance.

Let us indicate with *ω*_*ij *_the taxonomic distance between OTUs *i *and *j*, which are joined by *N *steps (branches) on the tree. Now we can define:

where *l*_*n *_is the length of the *n*th branch, *n = 1*, *2*, ..., *N*. We do not want to rely on information about mutation rates nor genetic distances. If we consider that a Linnean classification is mostly arbitrary, we can set branch lengths in several ways. Further considerations on this point are given above (Results; but see also [18]). The simplest case is considering a length equal to 1 for all branches. Accordingly, the distance between taxa 1 and 5 in Figure 8 is 4, and the distance between taxa 4 and 8 is 6. Indeed, taxa 1 and 4 are more closely related than taxa 4 and 8 are. The Average Taxonomic Distinctness (AvTD) of the tree is defined as the average of all such pairwise distances:

(modified from [26])

where *S *is the number of taxa in the tree. Given the presence/absence data case, and with the distance between taxa *i *and *j*, being *i = j*, set to 0 (same taxon), we note that the formula can be reduced to the computationally simpler form:

For example, the AvTD for the tree in Figure 8 would equal approximately 5.0556. The original formulation of the index considers also relative abundances of species, but here we only take into account presence/absence of OTUs.

This is the basic statistic described in this work. AvTD has been shown to be a good ecological indicator and a reliable estimator of biodiversity [37,41-43]. The most appealing feature is its clear independence from sampling effort ([36,37]; see Discussion above).

### Test of significance

The AvTD statistic simply gives the expected path length for a randomly selected pair of species from the set of *S *species [26]. The higher the AvTD, the more taxonomically distinct is the sample. However, it is necessary to compare the AvTD of a sample to the master list from which it is taken; for example, we may be interested in the molecular phylogeny of an order and we sampled and sequenced *S *species within this order. Naturally, we wish to maximize the number of families and genera represented therein. Using the AvTD method, we can estimate this "maximization" by computing the index for our sample of *S *species, and then comparing it with one computed from the list of all species belonging to the order itself. However, comparing a pure number to another pure number is rather uninformative; therefore, a random resampling approach to test for significance is suggested here. The rationale is as follows: we must estimate whether our sample's AvTD (AvTD_S_) is significantly different from the master list's one. Although the index is poorly dependent on sampling effort, we have to take into account that often the master list is consistently bigger than our sample. Thus, we draw *k *samples of size *S *from master list. We then compute AvTD from all *k *sample and test whether AvTD_S _falls within the 95% confidence limits of the distribution (original two-tailed test; but see Discussion above).

### Variation in Taxonomic Distinctness (VarTD)

As noted by Clarke and Warwick [40], some differences in the structure of the taxonomic trees of samples are not fully resolved by AvTD measures. Two taxonomic trees could have very different structures, in terms of subdivision of taxa into upper-level categories, but nevertheless could have the same AvTD. Differences in taxonomic structures of samples are well described by a further index of biodiversity, the Variation in Taxonomic Distinctness (VarTD).

VarTD is computed as a standard statistical variance. It captures the distribution of taxa between levels, and should be added to AvTD in order to obtain a good measure of biodiversity. Clarke and Warwick [26] demonstrated that VarTD can be estimated via a precise formula, but can also be obtained in the canonical statistical way from AvTD data.

Clarke and Warwick [40] proposed to follow the same procedure as above: observed VarTD is compared with values from random resamplings of the same size. Lower values of VarTD are preferable, as they are an indication of equal subdivision of taxa among intermediate levels. Clarke and Warwick [40] also show that VarTD is not as independent from sampling effort as AvTD is, i.e., there is a bias towards lower values for very small *S *(see Figure 2 and 4), but it can be shown [40] that this bias becomes rather negligible for *S >*10.

### Von Euler's index of imbalance

Following the idea of AvTD, von Euler [44] proposed an index related to taxonomic distinctness, which he called an *index of imbalance*. An index of imbalance measures the imbalance of the tree, i.e., whether and how much certain groups are under-represented and certain others are over-represented. This was not the first of such indexes [e.g., [45-48]]; however, as noted by Mooers and Heard [49], they do not apply to trees with polytomies, as taxonomic trees often are. Von Euler's index of imbalance (*I*_*E*_) is defined as:

where AvTD_max _and AvTD_min _are respectively the maximum and minimum possible AvTDs given a particular sample. AvTD_max _is obtained from a totally-balanced tree constructed on the given taxa, whereas AvTD_min _is obtained from a totally-imbalanced one.

**Figure 9:**
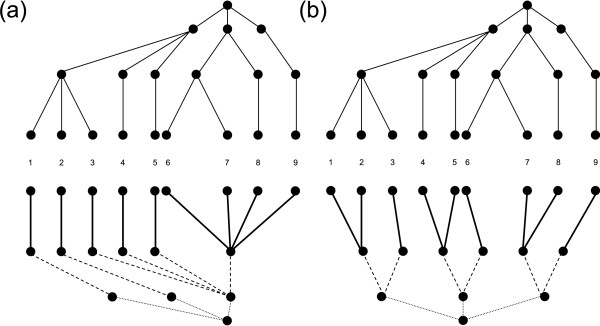
**Totally-imbalanced and totally-balanced taxonomic trees**. Totally-imbalanced (a) and totally-balanced (b) taxonomic trees computed starting from the taxonomic tree introduced in Figure 8 and shown at the top of both sides. See text for more details.

Figure 9 depicts such trees as computed from the taxonomic tree shown in Figure 8; taxonomic levels are considered as orders, families, genera, and species. (i) *Obtaining a completely imbalanced tree*. The procedure is bottom-up. Each species is assigned to a different genus (left side, thick lines, species 1, 2, 3, 4, and 5), until the number of "occupied" genera equals the total number of genera minus one. Remaining species are then lumped in the last genus (right side, thick lines, species 6, 7, 8, and 9). The same procedure is repeated in assigning genera to families (dashed lines). As we consider only one order, all families are lumped in it (dotted lines). More generally, the procedure is repeated until the uppermost hierarchical level is reached. (ii) *Obtaining a completely balanced tree*. The procedure is top-down. The first step is forced, as all Families must be lumped in the only present order (dotted lines). Then we proceed assigning (as far as possible) the same number of genera to each Family. In this case, we have 6 genera for 3 families, therefore it is very easy to see that the optimal distribution is 6/3 = 2 genera/family (dashed lines). The same step is repeated until the lowermost hierarchical level is reached. Each time we try to optimize the number of taxa which are assigned to all upper levels. We have in this case 9 species for 6 genera (thick lines). Necessarily we will have at best 3 genera with 2 species and 3 genera with 1 species (3 × 2 + 3 × 1 = 9). The optimal situation is the one depicted in the figure. For this reason, it is important to balance taxa not only with respect to the immediately upper taxon, but also with respect to all upper taxa. We note that the completely-balanced and completely-imbalanced trees may not be unique. However, differences in AvTD from different equally-balanced or equally-imbalanced trees are null or negligible.

As the original formulation of AvTD, von Euler's index of imbalance was introduced in the conservation context, since it was used to take estimates on the loss of evolutionary history, and was found to be strictly (negatively) correlated with AvTD (pers. obs.; [44]). We introduce *I*_*E *_in our topic, stating it is a useful balancing indicator for samples used in phylogenetic studies.

### Shuffling analysis

Shuffling analysis concepts and purposes are extensively explained in the Results section. Here we think it is useful to report algorithms that were written to carry it out, especially for shuffling phase.

#### Shuffling phase

User inputs the number of shuffled master lists they want to generate. The user must also decide the number of repetitions for each kind of move. Therefore, each of the following algorithms is repeated the given number of times on the same master list. Then, the resulting file is saved to disk and a new one is produced, with same modalities.

Move: **Transfer**

1. user is requested to input a taxon level *t*, with *t = 1*, *2*, ..., *T - 1*;

2. a taxon *a *of level *t *is randomly chosen;

3. *if *taxon *A *of level *t + 1 *containing *a *contains only *a*

*then *return to 2;

*else *proceed to 4;

4. a taxon *B *of level *t + 1 *is randomly chosen;

5. *if *taxon *B *= taxon *A*

*then *return to 4;

*else *proceed to 6;

6. taxon *a *is moved to taxon *B*.

Move: **Split**

1. user is requested to input a taxon level *t*, with *t = 2*, ..., *T - 1*;

2. a taxon *a *of level *t *is randomly chosen;

3. taxon *a *is split into two new taxa in the same position.

Move: **Merge**

1. user is requested to input a taxon level *t*, with *t = 2*, ..., *T - 1*;

2. a taxon *a *of level *t *is randomly chosen;

3. *if *taxon *A *of level *t + 1 *containing *a *contains only *a*

*then *return to 2;

*else *proceed to 4;

4. a taxon *b *of level *t *is randomly chosen within taxon *A*;

5. *if a = b*

*then *return to 4;

*else *proceed to 6;

6. taxa *a *and *b *are merged in a new taxon in the same position.

In all moves, downstream relationships are maintained. For example, if genus *a *containing species *α *and *β *is moved from family *A *to family *B*, species *α *and *β *will still belong to genus *a *within family *B*. The same holds true for splits and merges.

#### Analysis phase

In this phase, the basic phylogenetic representativeness analysis is applied on each master list. Therefore, a large number (depending upon the chosen number of master lists to be simulated) of analyses are performed and consequently six sets of measurements are obtained for each dimension *s*, namely the six parameters describing AvTD and VarTD:

lower AvTD 95% confidence limit;

mean AvTD;

mean VarTD;

upper VarTD 95% confidence limit;

maximum AvTD;

minimum VarTD;

For the first four sets of measurements, upper and lower 95% confidence limits are computed for each dimension *s *across all master lists, thus giving an idea of the stability of results. For the fifth and sixth sets of measurement, simply the maximum entry is kept for each dimension *s *as above.

## Authors' contributions

FP conceived the study and developed the Clarke and Warwick's statistics in a phylogenetic framework. RRF wrote the PhyRe software and helped to draft the manuscript. MP participated in designing and coordinating this study, and provided many essential comments. All authors read and approved the final manuscript.

## Supplementary Material

Additional file 1 **PhyRe scripts and documentation**. Three Python scripts constitute the PhyRe package. PhyRe script itself performs main analyses presented in this paper: AvTD, VarTD, I_E_, and funnel plots parameters are computed by this script. PhyloSample generates shuffled master lists, whereas PhyloAnalysis repeats PhyRe tasks across all newly-generated master lists. All scripts have been tested under Python 2.5.4. PhyRe documentation (doc.pdf) and eight sample files referring to datasets used in the paper to validate the method [19-22] are also enclosed. Click here for file

Additional file 2 **Real and simulated data from bivalve data set**. Real and simulated data from bivalves data set follow Millard [23] reference taxonomy. Table shows the composition of our real and simulated samples of bivalves. Taxonomy is reported for each genus; a plus "+" sign indicates the presence of that genus in that sample. Click here for file
