# Supplementary material for: Phylogenetic representativeness: a new method for evaluating taxon sampling in evolutionary studies
Source: BMC Bioinformatics. 2010 Apr 27;11:209. doi: 10.1186/1471-2105-11-209 (PMC2871275; doi:10.1186/1471-2105-11-209)
Supplement: Additional file 1 — PhyRe scripts and documentation. Three Python scripts constitute the PhyRe package. PhyRe script itself performs main analyses presented in this paper: AvTD, VarTD, IE, and funnel plots parameters are computed by this script. PhyloSample generates shuffled master lists, whereas PhyloAnalysis repeats PhyRe tasks across all newly-generated master lists. All scripts have been tested under Python 2.5.4. PhyRe documentation (doc.pdf) and eight sample files referring to datasets used in the paper to validate the method [19-22] are also enclosed. [file 1471-2105-11-209-S1.ZIP › doc.pdf]

PhyRe

Phylogenetic Representativeness

V1.0

Proper citation: Plazzi, F., R. R. Ferrucci, and M. Passamonti. 2009. XXX

DISCLAIMER

Copyright © 2009 Ronald R. Ferrucci, Federico Plazzi, and Marco Passamonti.

Permission is hereby granted, free of charge, to any person obtaining a copy of this software and associated documentation files (the "Software"), to deal in the Software without restriction, including without limitation the rights to use, copy, modify, merge, publish, distribute, sublicense, and/or sell copies of the Software, and to permit persons to whom the Software is furnished to do so, subject to the following conditions:

The above copyright notice and this permission notice shall be included in all copies or substantial portions of the Software.

THE SOFTWARE IS PROVIDED "AS IS", WITHOUT WARRANTY OF ANY KIND, EXPRESS OR IMPLIED, INCLUDING BUT NOT LIMITED TO THE WARRANTIES OF MERCHANTABILITY, FITNESS FOR A PARTICULAR PURPOSE AND NONINFRINGEMENT. IN NO EVENT SHALL THE AUTHORS OR COPYRIGHT HOLDERS BE LIABLE FOR ANY CLAIM, DAMAGES OR OTHER LIABILITY, WHETHER IN AN ACTION OF CONTRACT, TORT OR OTHERWISE, ARISING FROM, OUT OF OR IN CONNECTION WITH THE SOFTWARE OR THE USE OR OTHER DEALINGS IN THE SOFTWARE.

CORRESPONDING AUTHOR

Questions, issues, or suggestions regarding the software should be directed to R. R. Ferrucci: ronaldrobert dot ferrucci at unife dot it.

Index

1. Theoretical framework 4

1.1. PHYLOGENETIC REPRESENTATIVENESS. 4

1.2. MASTER LISTS. 4

1.3. PATH LENGTHS. 5

1.4. FUNNEL PLOTS. 5

1.5 SHUFFLING ANALYSES. 6

2. Installing and running PhyRe 7

3. PhyRe 7

3.1. INPUT FILES. 7

3.2. SETTING UP THE ANALYSES. 9

3.2.1. FUNNEL PLOTS. 9

3.2.2. MISSING DATA. 9

3.2.3. CUSTOM PATH LENGTHS. 10

3.2.4. OUTPUT. 10

3.3. BATCH JOBS. 11

3.4. OUTPUT FILES. 12

4. PhyloSample 13

4.1. INTERACTIVE MODE. 14

4.2. AUTOMATED MODE. 15

5. PhyloAnalysis 16

6. Bibliography 19

1. Theoretical framework

1.1. Phylogenetic Representativeness.

Phylogenetic Representativeness is a method for estimating adequacy of taxon sampling for phylogenetic studies. Through a series of statistics, it has become possible to evaluate taxon coverage within a given ingroup. The method relies entirely on taxonomic information and is based on ecological indexes (Warwick and Clarke, 1995; Clarke and Warwick, 1998; Warwick and Clarke, 1998; Clarke and Warwick, 2001; von Euler, 2001). Basically, two taxonomic trees are analyzed. A taxonomic tree is a graphical representation of a standard Linnean classification. The first taxonomic tree shows taxonomic placement of the sample which is to be evaluated. The second one refers to a master list, *i.e.* the complete list of the group which the sample should represent. A measure of distance (called "taxonomic distance", as it does not involve any genetical or *a priori* phylogenetical information) is taken for each pair of OTUs on the trees. The mean taxonomic distance is the Average Taxonomic Distinctness (AvTD) of that tree. The variance is the Variation in Taxonomic Distinctness (VarTD). These statistics from the sample must be compared to the same statistics estimated on the master list. This is done by assessing confidence limits. *k* subsamples of varying sizes are randomly drawn from the master list and a "funnel plot" is constructed. As high values of AvTD are obtained from samples with high distinctness, we say a sample is representative of a population if it is comprehended within the lower 95% confidence limit for its own dimension *S*. Conversely, low values of VarTD are preferable, as this means the sample is equally dispersed on each intermediate-level taxon. Thus, a sample must fall within the upper 95% confidence limit in VarTD funnel plots to be representative. A third statistic is the von Euler Index of Imbalance (I_E), which gives a rough idea about under- or over-representation of groups within a given sample. The method is extensively illustrated in the paper by Plazzi et al., (XXX).

1.2. Master lists.

Master lists can include both extant and fossil taxa. Any taxonomic level may be considered, from classical Linnean levels to intermediate ones (such as Infraclasses, Superfamilies, Sections, etc.). For the moment, PhyRe does not implement population level analyses, meaning that indexes' versions corrected for relative abundance within

taxa are not implemented yet. Nevertheless, Phylogenetic Representativeness can potentially work also for at the population level. However, it is not required that terminal leaves correspond to species: they may correspond to Genera, subspecies, or other.

1.3. PATH LENGTHS.

On taxonomic trees, path lengths are substantially arbitrary. However, it is preferable to rescale all path lengths in a way that they sum to some fixed value, generally set to 100. In this way, adding or removing taxonomic levels does not affect the validity of previous analyses (Clarke and Warwick, 1999). Step lengths can be weighted all the same. Otherwise, they can be weighted proportionally to the loss of biodiversity at each step. Clarke and Warwick (1999) have shown that the method of step weighting does not affect results.

1.4. FUNNEL PLOTS.

Funnel plots show four kinds of information. These are:

- AvTD or VarTD values for experimental samples;
- 95% confidence limits, which is the lower one for AvTD plots and the upper one for VarTD plots;
- mean AvTD or VarTD values computed from all random subsamples;
- the maximum (or the minimum) value of AvTD (VarTD) found across all replicates.

Experimental samples must fall between the confidence limit and the opposite extreme value to be considered representative of the population. It is also possible for some samples to be plotted above the maximum limit of AvTD or below the minimum limit

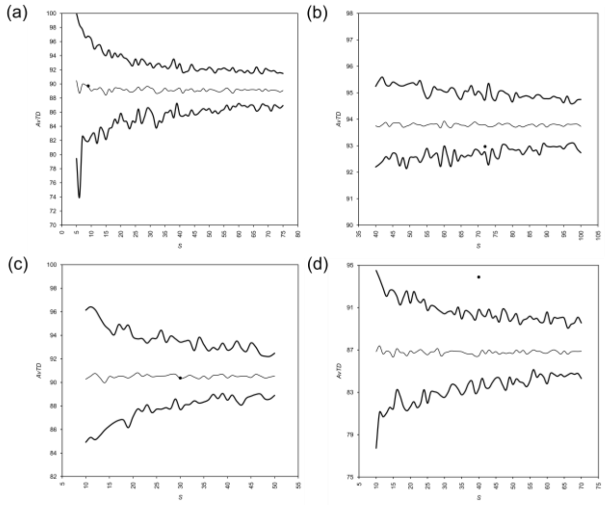

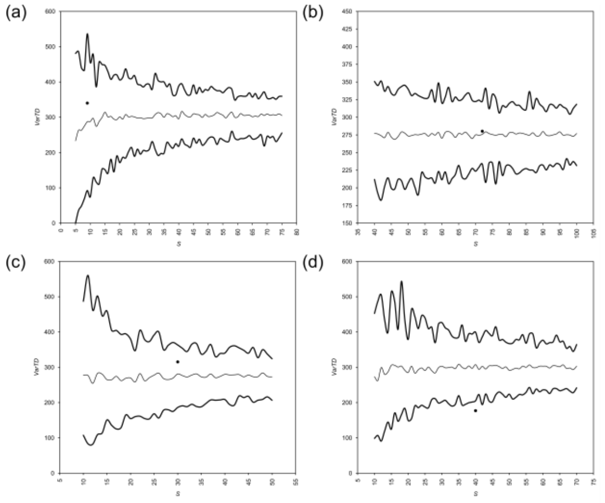

of VarTD, because not necessarily, given a finite number of replicates, the software will find the most extreme possible value. These are samples that are strikingly representative and informative about their group. Shown is an example taken from Plazzi et al. (XXX), which shows AvTD and VarTD funnel plots from four published data sets – a, bivalves (Passamaneck, et al., 2004); b, carnivores (Flynn et al., 2005); c, coleoids (Strugnell et al., 2005); d, termites (Legendre et al., 2008).

1.5. Shuffling analysis.

Taxonomies are human projections on reality, in the sense that most taxa and hierarchical rankings we deal with are not actually existing as concrete objects. Therefore, taxonomy is necessarlily on a large extent subjective, and this may bias our method, which is entirely based on them. Nonetheless, we think that hundreds of years of work made extant taxonomies largely stable, albeit for most eukaryotic groups: widespread agreement has been reached on high-level classification of most phyla, and wide-scale rearrangements seem unlikely now. However, revisions and improvements are continuously added, and it is worthy testing how could a possible modification in the underlying taxonomy change outcomes from our tests: two scripts were written appositely for this function and our procedure can essentially be described in two phases. In the first one, the shuffling phase, master lists are shuffled, resulting in a large number of alternative master lists. In the second, the analysis phase, a phylogenetic representativeness analysis is carried out as described above across all simulated master lists rearrangements.

The shuffling phase is composed of three changes, which are repeated and combined *ad libitum*: (1) a taxon is randomly transferred to another upper-level taxon; (2) a taxon is merged to a sister group; or (3) a taxon is split into two sister groups. Each move is separately repeated, randomly involving different taxa, a given number of times, until a new master list is produced. These moves simulate the commonest operations taxonomists do when reviewing a classification. A large number of “reviewed” master lists is then produced, repeating each time the same numbers of moves. Finally, the shuffling phase ends with a set of master lists.

Standard phylogenetic representativeness analyses are performed on each master list, and all statistics are computed for each list. In this way, a set of measurements are produced for each indicator. Therefore, it is possible to compute standard 95% (two-tailed) confidence intervals for each one. This analysis phase gives an idea of the funnel plot's width of oscillation upon revision.

2. Installing and running PhyRe

PhyRe scripts are available as Python source codes; the main script is also available as independent Windows executable file. PhyRe scripts has been tested on python 2.5.4; please remember that: (1) Python is case-sensitive; (2) scripts will always overwrite without asking existing output files if they are already present. It is easiest to run the program with input files placed into the same directory as the program.

The syntax for Python scripts or Windows executable files is the same, the only difference being that Python installation can be avoided in the latter case and either **`python scriptname.py`** or **`scriptname.exe`** must be typed, respectively, as shown below.

3. PhyRe

PhyRe must be run from the command line by typing:

python PhyRe.py [samplefile] [masterlistfile] s_1 s_2 [options]

Alternatively,

PhyRe.exe [samplefile] [masterlistfile] s_1 s_2 [options]

runs the standalone Windows executable.

3.1 Input files.

PhyRe requires two input files: a sample file and a population file. The sample file is the file describing the experimental sample: Phylogenetic Representativeness will be estimated for this sample. The population file is a file containing the master list of organisms; in other words, the sample file must be subset of the master list. As PhyRe will not behave correctly if taxa included in the sample file are not present in the population file, the user should be careful on this point.

PhyRe takes as input a plain text file, without regard to its extension. Each line must contain one OTU. Taxonomic levels are separated by spaces or tabs and must not contain whitespace within levels, which must be replaced with commas, underscores, hyphens, or

other non-whitespace character (or just removed). It may be useful to export a spreadsheet as a tab delimited text to obtain an input file for PhyRe. The first column must contain a “name” for the row; subsequent columns must list taxonomic level, from upper to lower. The up-most level, the one including all others (i. e., the root of the taxonomic tree), must not be included in the file. Generally, the last column will be identical to the first one, unless the user does not identify their OTUs with custom names.

Master lists can be rather difficult to find, depending upon the group of interest. Often there are complete checklist available online, as in the case of mammals (compiled by Robert B. Hole, Jr.; http://www.interaktv.com/MAMMALS/Mamtitl.html; and reference therein). Otherwise, they must be manually compiled or adjusted from some sort of electronic format. For example, the taxonomic compendium of mollusks by Millard (2001) encloses a PDF containing the entire master list for mollusks.

In the event that the master file contains duplicate samples, these will be ignored in the analyses, but will be included in the output. Duplicates are determined by duplicate sample names; therefore, they may be the same, in terms of taxa at each level, or may just have identical sample names but taxonomic differences. In the case of duplicates, the user is recommended to investigate the duplicates in the master list to ensure correct input.

Population file must contain one header starting with word “Taxon” followed by a colon “:” and list taxonomic levels to be used for the analyses, again from upper to lower. A second header, which is not mandatory, may be included: it is used to specify user-defined path lengths if desired by the user. By default, PhyRe computes path lengths from biodiversity loss across levels. A header starting with “Coefficients” followed by a colon “:” can define step lengths, from upper to lower steps. The sum does not necessarily have to sum to 100, but a rescaling such as this is strongly encouraged. It is important to verify that the number of step lengths equals the number of taxa, as each step length here is the sum of the two identical branches which start from and arrive to a given level. Both headers can be present also in the sample file (if useful to track analysis) and they will be ignored by PhyRe.

Below is reported, as an example, the first part of carnivore population file, which is distributed along with the package. A “**`Coefficients:`**” header has been added. A sample file is identical, but it does not have any header and contains only lines referring to taxa present in that sample. Tabs are not required for spacing taxa, but are used for clarity.

```
Taxon: family subfamily genus species
Coefficients: 25 25 25 25
Alopex_lagopus              Canidae      /      Alopex        Alopex_lagopus
Atelocynus_microtis         Canidae      /      Atelocynus    Atelocynus_microtis
Canis_adustus               Canidae      /      Canis         Canis_adustus
Canis_aureus                Canidae      /      Canis         Canis_aureus
Canis_latrans               Canidae      /      Canis         Canis_latrans
Canis_lupus                 Canidae      /      Canis         Canis_lupus
Canis_mesomelas             Canidae      /      Canis         Canis_mesomelas
Canis_rufus                 Canidae      /      Canis         Canis_rufus
Canis_simensis              Canidae      /      Canis         Canis_simensis
Cerdocyon_thous             Canidae      /      Cerdocyon     Cerdocyon_thous
Chrysocyon_brachyurus       Canidae      /      Chrysocyon    Chrysocyon_brachyurus
Cuon_alpinus                Canidae      /      Cuon          Cuon_alpinus
Dusicyon_australis          Canidae      /      Dusicyon      Dusicyon_australis
Lycaon_pictus               Canidae      /      Lycaon        Lycaon_pictus
Nyctereutes_procyonoides    Canidae      /      Nyctereutes   Nyctereutes_procyonoides
```

3.2. SETTING UP THE ANALYSES.

3.2.1. FUNNEL PLOTS.

PhyRe does not draw funnel plots, but it formats results in such a way that it is very easy to copy and paste them to a graph-drawing software, like Microsoft Excel®. Immediately after sample and population file, PhyRe expects **`s1`** and **`s2`**, i. e. the lower and upper boundary of the subsample dimension range.

A custom number of permutation (default is 1,000) can be invoked by

`-p k` (="permutations")

3.2.2. MISSING DATA.

First of all, it is important to know whether there is missing data present in either input file. By default, PhyRe does not consider missing data. However, they are frequent, because many intermediate-levels are defined only in some cases within a given group. For example, subfamilies may have been established for just one family within a given order. In such cases, PhyRe can "fill" in the "empty field" copying the first not-blank upper taxonomic level. For example, again from the carnivore data set:

```
Odobenus_rosmarus           Odobenidae   /             Odobenus       Odobenus_rosmarus
Callorhinus_ursinus         Otariidae    /             Callorhinus    Callorhinus_ursinus
Arctocephalus_gazella       Otariidae    /             Arctocephalus  Arctocephalus_gazella
Zalophus_californianus      Otariidae    /             Zalophus       Zalophus_californianus
```

will become

```
Odobenus_rosmarus          Odobenidae   Odobenidae   Odobenus       Odobenus_rosmarus
Callorhinus_ursinus        Otariidae    Otariidae    Callorhinus    Callorhinus_ursinus
Arctocephalus_gazella      Otariidae    Otariidae    Arctocephalus  Arctocephalus_gazella
Zalophus_californianus     Otariidae    Otariidae    Zalophus       Zalophus_californianus
```

Odobenus rosmarus and *Callorhinus ursinus* would have been recognized as being in the same subfamily (named "**/**"); with this correction, they correctly appear in two different subfamilies, because now "Odobenidae" is a different string with respect to "Otariidae".

This is accomplished by adding the option:

-m y (="missing data yes")

This option recognizes only "**/**" as a missing data character and needs the uppermost taxon to be specified. If not present, the user must manually input the taxon; PhyRe will then copy that string if necessary.

3.2.3. CUSTOM PATH LENGTHS.

If desired, the "**Coefficients:**" header will be read if the user sets the option

-l y (= "[custom] lengths yes")

Output file will list the enforced path lengths. PhyRe will not, however, check whether the sum is 100.

3.2.4. OUTPUT.

Two output files are printed: the first shows results from analyses of the sample, whereas in the second one users will find results from random subsamples of the master list. By default, the output file has the same name of the sample file with extension .OUT, whereas the funnel output file has the same name with suffix "_funnel" and extension .OUT. It is possible to specify a custom name with option

-o [name] (="output file")

For example, by using

-o carnivores

PhyRe will write sample's result to a **carnivores.out** file and funnel plot statistics to a **carnivores_funnel.out** file.

It is also possible to override the computation of the funnel plot and have only result from a single sample, by the option

-c n (="confidence limits no")

This option may be desired in the event that the user wishes to analyze additional data after running PhyRe using the same master list, and therefore has no need for additional output of funnel plot data. PhyRe will however anyway require a master list for its correct working.

3.3. Batch jobs.

It is possible to test more than a sample at once, by saving samples to separate input files and providing PhyRe with sample file listing them. No header must be included in the batch file and the following option must be set to yes

-b y (="batch file yes")

Below is reported an example of a batch file, telling PhyRe to analyze samples called "**carnivores1.dat**", "**carnivores2.txt**", and "**carnivores3.txt**", that could correspond to different stages of a work or to different taxon samplings in the group. Such an analysis is strated by typing

python PhyRe.py [batchfile] [masterlistfile] [options]

```
carnivores1.dat
carnivores2.txt
carnivores3.txt
```

All sample files must be in the program folder, but they may have different extensions. All results will be separately written in a single output file. A unique funnel plot will also be computed.

3.4. OUTPUT FILES.

Below is reported an output file, as obtained from the dataset of carnivores, along with a sample from the funnel output file, which naturally can vary from analysis to analysis due to random resamplings. In this case, we typed at the shell:

python PhyRe.py carnivores_sample.txt carnivores_list.txt 40 100 –m y –p 100

```
Output from Average Taxonomic Distinctness

Carnivora_sample.txt
Number of taxa and path lengths for each taxonomic level:
family         11      32.7421
subfamily      23      18.7911
genus          129     29.5948
species        271     18.8720

---------------------------------------------------
Results for sample:  Carnivora_Flynn
Dimension for this sample is 72

Number of taxa and pairwise comparisons  at each taxon level:
family         11      4268
subfamily      25      490
genus          60      298
species        72      56

Number of pairwise comparisons is for pairs that differ at each level excluding comparisons that
differ at upper levels

Average taxonomic distinctness      = 92.9688
Variation in taxonomic distinctness = 280.2311
Minimum taxonomic distinctness      = 66.5806
Maximum taxonomic distinctness      = 96.5774
von Euler's index of imbalance      = 0.1203

---------------------------------------------------

Confidence limits for average taxonomic distinctness and variation in taxonomic distinctness
limits are lower 95% limit for AvTD and upper 95% limit for VarTD

Number of permutations for confidence limits = 100

dimension AvTD05%   AvTDmean  AvTD95%   AvTDup    VarTDlow   VarTD05%   VarTDmean  VarTD95%
40        92.1994   93.7470   95.0093   95.2429   211.9107   222.5909   277.2419   350.6591
41        92.2932   93.7177   95.0746   95.4592   192.0796   223.7237   276.3060   344.9892
42        92.4140   93.7960   95.2618   95.5888   182.5738   211.1686   273.8004   351.2555
43        92.5873   93.8803   95.0846   95.3075   201.9509   216.5352   271.1420   336.8609
```

44	92.4964	93.7240	94.9145	95.2600	214.2142	228.9437	278.6014	343.5613
[…]								
96	93.0308	93.8119	94.4702	94.8702	225.9509	243.6532	274.7120	314.6410
97	93.0989	93.8044	94.3879	94.6034	241.3172	246.2888	275.2682	311.8376
98	93.0866	93.8147	94.5183	94.6075	232.2756	247.3679	274.8236	304.3116
99	92.8703	93.8124	94.4313	94.7285	237.2272	246.0144	272.9568	312.5469
100	92.7367	93.7311	94.4425	94.7464	231.5224	247.7170	276.9281	318.4531

For each dimension, funnel plot output includes: lower 95% confidence limit, mean, upper 95% confidence limit, and absolute upper limit for AvTD; absolute lower limit, lower 95% confidence limit, mean, and upper 95% confidence limit for VarTD. The confidence limits are actually one-tailed, with the important, statistical significance limits being the lower and upper 95% limits for AvTD and VarTD, respectively. Upper and lower 95% confidence limits for AvTD and VarTD, respectively, are provided for proper funnel shape production and have no bearing on statistical significance. Absolute lower and upper limits for AvTD and VarTD, respectively, are provided for boundary identification.

Data regarding funnel plots must be plotted along with results from the first file, which tells us that the AvTD for this sample is 92.9530, VarTD is 282.261, and I_E is 0.1200 (underlined; the underlining is of the manual).

4. PhyloSample

PhyloSample performs the shuffling phase of test on master list stability. It must be run from the command line by typing:

```
python PhyloSample.py [masterlistfile] [outputfile] [options]
```

This script will take a master list as input (formatted exactly as above) and create two folders, namely **[outputfile]MasterChanges** and **[outputfile]MasterFiles**. In the former folder, **.changes** files will be stored; in the latter one, **.sim** files will be stored. For each repetition, these extensions denote a file extensively listing which taxa were drawn for each move and the new master list, respectively.

Each file will also be numbered, so that, for example, if **[outputfile]** is set **[carnivores_shuffling]**,

```
carnivores_shuffling_43.sim
```

will denote the forty-third master list produced from original data and will be found in a folder called **carnivores_shufflingMasterFiles**.

Basically, two ways are present for shuffling master lists with PhyloSample: the interactive and the automated mode.

4.1. INTERACTIVE MODE.

To invoke the interactive mode, the command line input is simply

`python PhyloSample.py [masterlistfile] [outputfile] i [-m y]`

PhyloSample will read the population file, and then ask user

`what function would you like to perform? input m for move, e for merge, or s for split`

User must choose the move to be performed by typing the relative letter and pressing the enter key. The program will then ask the taxonomic rank to be affected by changes (identifying each level, as read from the "**`Taxon:`**" header, with a number), the number of taxa to merged together in the merging case, and the number of repetitions, i. e. how many times such a taxon must be drawn from the original master list and manipulated as chosen. Then the question

`would you like to continue? please input y for yes or n for no`

will appear. If "**`n`**" is chosen, the **`.changes`** and **`.sim`** file are created, and the program exits. Otherwise, changes are retained in memory and new moves can be implemented on the same data set. When enough moves have been performed, the new master list can be saved. Please note that the interactive mode creates just one shuffled master list and, therefore, does not create two folders and does not add number to file names: new files are placed in the same directory where the original master list is. Folders must be manually created and files must be manually moved within them; if a second shuffled list is to be generated, either a different **`[outputfile]`** option must be used, or the number must be manually added.Interactive mode is not included for a true shuffling analysis, which typically involves the generation of at least 100 master lists – this would be awkward. This mode was provided for practice with shuffling phase and for special cases: indeed, only in the interactive mode it is possible to perform moves on different taxonomic

levels and merge more than two taxa. The missing data option can be enforced at the end of the input line to take into account them during analyses. See above for details.

4.2. Automated mode.

The automated move syntax is

```
python PhyloSample.py [masterlistfile] [outputfile] a n t s e m [-m y]
```

Options are as follows.

- **`a`**
  Tells to PhyloSample to run in automated mode.
- **`n`**
  Number of master lists to be generated. In automated mode, PhyloSample creates folders and numerates files as described above.
- **`t`**
  The taxon level to work on. This must be provided exactly as it is written in the "**`Taxon:`**" header in the population file, and not as a number as in interactive mode for example, "**`family`**" or "**`subfamily`**" – without quotes). All moves for all master lists will be performed on this taxon level only.
- **`s`**
  The number of split moves to be performed. Please note that PhyloSample can not split lowest-level taxa: the original name would be replaced by two new ones and, if a taxon that is also included in the sample file is drawn, the relation between population and sample file would be lost.
- **`e`**
  The number of merge moves to be performed. Please note that PhyloSample can not merge lowest-level taxa: the original names would be replaced by a new one and, if a taxon that is also included in the sample file is drawn, the relation between population and sample file would be lost. Automated move will always merge two taxa.
- **`m`**
  The number of move moves to be performed. Lowest-level taxa can be moved without side-effects.
- **`[-m y]`**
  Again, the missing data option can be enforced at the end of the input line to take into account them during analyses.

PhyloSample will generate the requested number of master lists and put files in their own folders; then the program exits. The progress can be tracked by checking how many master lists have been saved to the MasterFiles folder. Please note that **`.changes`** files are not useful in subsequent analyses: they are provided to accurately check PhyloSample operations in case it is needed. As each move is set to involve different random taxa, PhyloSample jobs can take a long time if many moves are to be repeated when there are few taxa at the chosen taxonomic level: this makes unlikely the drawing of a different taxon for each move, and therefore the software may get trapped waiting to draw a new taxon to continue shuffling. Please be careful in shuffling tuning to overcome this issue.

5. PhyloAnalysis

PhyloAnalysis is provided to perform PhyRe analysis on all master lists produced by PhyloSample. The syntax for this script is

python PhyloSample.py [masterlistsfolder] [outputfile] p s_1 s_2 [options]

This will tell to the software where to find master lists; typically, this folder will have a name like "**`Carnivores_shufflingMasterFiles`**", as it was created by PhyloSample. The script will look for files having all the same name, but the final number. Results from PhyloAnalysis will be printed out in a file called **`[outputfile].psa`**; a log (**`[outputfile].txt`**) will also be produced to track the progress. Options are the same as for PhyRe (see above): s_1 and s_2 are lower and upper boundaries for funnel plot characterization, whereas **p** is the number of subsamples to be drawn from each master list.

Below the first few lines of a typical log file are reported.

```
2009-11-02 22:20:53,645 INFO begin analysis of master lists
2009-11-02 22:20:53,653 INFO master list: Carnivora_shuffling_1.sim
2009-11-02 22:20:53,723 INFO dimension: 40
2009-11-02 22:20:53,723 INFO permutation: 1
2009-11-02 22:20:53,726 INFO permutation: 2
2009-11-02 22:20:53,729 INFO permutation: 3
2009-11-02 22:20:53,732 INFO permutation: 4
2009-11-02 22:20:53,733 INFO permutation: 5
2009-11-02 22:20:53,736 INFO permutation: 6
2009-11-02 22:20:53,740 INFO permutation: 7
2009-11-02 22:20:53,743 INFO permutation: 8
2009-11-02 22:20:53,744 INFO permutation: 9
```

```
2009-11-02 22:20:53,747 INFO permutation: 10
2009-11-02 22:20:53,750 INFO permutation: 11
2009-11-02 22:20:53,753 INFO permutation: 12
2009-11-02 22:20:53,755 INFO permutation: 13
2009-11-02 22:20:53,757 INFO permutation: 14
2009-11-02 22:20:53,759 INFO permutation: 15
```

This means that PhyloAnalysis has completed the fifteenth subsampling of dimension 40 on the first master list. Provided time on the left make prevision possible on the computational time required to complete the work; please note that PhyloAnalysis may take a long time to complete the job, especially when a large number of master lists is analyzed.

Allowed options are:

- **`-m y`**
  for population files with missing data.
- **`-l y`**
  for use with custom path lengths.

An error may be returned when low numbers of master lists are analyzed for a low number of repetitions; therefore, we suggest to always analyze at least 100 subsamplings from 100 master lists.

As for PhyRe, the output file will provide all statistics in a tabular format, so that it is easy to import them into a spreadsheet software for graph drawing. Six parameters describing AvTD and VarTD are computed and printed:

- lower AvTD 95% confidence limit;
- mean AvTD;
- mean VarTD;
- upper VarTD 95% confidence limit;
- maximum AvTD;
- minimum VarTD;

For the first four sets of measurements, upper and lower 95% confidence limits are computed for each dimension **`s`** across all master lists, thus giving an idea of the stability of results. For the fifth and sixth sets of measurement, simply the maximum entry is kept for each dimension **`s`** as above. Therefore, the output file will look like the one displayed in the next page, quite similarly to above. Please note that 2.5% and 97.5% confidence limit are not estimated for Maximum AvTD and minimum VarTD, as these are only descriptive measures, useful to track the shape of the funnel plot. Stability of taxonomies is addressed by carefully examining funnel plots obtained from the eight other measures.

d	AvTD05%_2.5%	AvTD05%_97.5%	AvTDmean_2.5%	AvTDmean_97.5%	AvTDmax	VarTD95%_2.5%	VarTD95%_97.5%	VarTDmean_2.5%	VarTDmean_97.5%	VarTDmin
40	90.6820	91.3418	92.7298	93.1534	95.9657	404.6123	425.4087	310.3569	319.2997	165.2475
41	90.6388	91.3780	92.6014	93.1395	95.9592	402.1000	425.5932	308.3345	319.8813	166.0856
42	90.8198	91.4076	92.6040	93.1513	95.8908	398.3016	420.9783	309.5619	318.6990	173.3310
43	90.7084	91.4803	92.7173	93.1566	95.9448	400.8057	420.2643	310.7866	319.5751	177.7508
44	90.8111	91.5014	92.6866	93.1544	95.9413	395.7380	418.9847	311.7081	320.1982	166.2407
[…]										
96	91.8179	92.2920	92.7171	93.1643	95.2004	368.6690	379.5266	314.5664	322.5723	223.0617
97	91.8496	92.3236	92.6631	93.1567	95.2470	367.7593	378.8356	316.0720	322.5663	219.1732
98	91.8425	92.3088	92.7053	93.1694	95.1994	366.9206	377.8576	315.9899	323.0449	225.0649
99	91.8675	92.3152	92.6937	93.1683	95.2293	368.8502	377.6154	316.0566	322.4042	222.0376
100	91.8089	92.3067	92.6693	93.1660	95.2172	365.6926	376.9409	316.0978	322.3787	225.9684

6. Bibliography

Clarke, K. R., and R. M. Warwick. 1998. A taxonomic distinctness index and its statistical properties. J. Appl. Ecol. 35:523-531.

Clarke, K. R., and R. M. Warwick. 1999. The taxonomic distinctness measure of biodiversity: Weighting of step lengths between hierarchical levels. Mar. Ecol. Prog. Ser. 184:21-29.

Clarke, K. R., and R. M. Warwick. 2001. A further biodiversity index applicable to species lists: Variation in taxonomic distinctness. Mar. Ecol. Prog. Ser. 216:265-278.

Flynn, J. J., J. A. Finarelli, S. Zehr, J. Hsu, and M. A. Nedbal. 2005. Molecular phylogeny of the Carnivora (Mammalia): Assessing the impact of increased sampling on resolving enigmatic relationships. Syst. Biol. 54:317-337.

Legendre, F., M. F. Whiting, C. Bordereau, E. M. Cancello, and T. A. Evans. 2008. The phylogeny of termites (Dictyoptera: Isoptera) based on mitochondrial and nuclear markers: Implications for the evolution of the worker and pseudergate castes, and foraging behaviors. Mol. Phylogenet. Evol. 48:615-627.

Millard, V. 2001. Classification of Mollusca: A classification of world wide Mollusca, 2nd edition. Vol. 3, Pages 915-1447. Printed by the author, South Africa.

Passamaneck, Y. J., C. Schlander, and K. M. Halanych. 2004. Investigation of molluscan phylogeny using large-subunit and small-subunit nuclear rRNA sequences. Mol. Phylogenet. Evol. 32: 25-38.

Plazzi, F., R. R. Ferrucci, and M. Passamonti. XXX

Strugnell, J., M. Norman, J. Jackson, A. J. Drummond, and A. Cooper. 2005. Molecular phylogeny of coleoid cephalopods (Mollusca: Cephalopoda) using a multigene approach; the effect of data partitioning on resolving phylogenies in a Bayesian framework. Mol. Phylogenet. Evol. 37:426-441.

von Euler, F. 2001. Selective extinction and rapid loss of evolutionary history in bird fauna. Proc. R. Soc. Lond. B 268:127-130.

Warwick, R. M., and K. R. Clarke. 1995. New “biodiversity” measures reveal a decrease in taxonomic distinctness with increasing stress. Mar. Ecol. Prog. Ser. 129:301-305.

Warwick, R. M., and K. R. Clarke. 1998. Taxonomic distinctness and environmental assessment. J. Appl. Ecol. 35:532-543.
