# Supplementary material for: Phylogenetic representativeness: a new method for evaluating taxon sampling in evolutionary studies
Source: BMC Bioinformatics. 2010 Apr 27;11:209. doi: 10.1186/1471-2105-11-209 (PMC2871275; doi:10.1186/1471-2105-11-209)
Supplement: Additional file 2 — Real and simulated data from bivalve data set. Real and simulated data from bivalves data set follow Millard [23] reference taxonomy. Table shows the composition of our real and simulated samples of bivalves. Taxonomy is reported for each genus; a plus "+" sign indicates the presence of that genus in that sample. [file 1471-2105-11-209-S2.PDF]

Additional File 1. Real and simulated data from bivalves dataset, following Millard (2001) reference taxonomy.

Table showing the composition of our real and simulated samples of bivalves. Taxonomy is reported for each Genus; a plus "+" sign indicates the presence of that Genus in that sample.

Subclass	Order	Family	Genus	R1	R2	R3	R4	S1	S2	S3	S4
HETERODONTA	CHAMIDA	MACTRIDAE	*ALIOMACTRA* Stephenson, 1952 [1953]							+	
HETERODONTA	VENEROIDA	CARDITIDAE	*AMEKIGLANS* Eames, 1957							+	
HETERODONTA	VENEROIDA	CONDYLOCARDIIDAE	*AMERICUNA* Klappenbach, 1962							+	
PTERIOMORPHIA	ARCIDA	ARCIDAE	*ANADARA* Gray, 1847	+	+	+	+				+
PALAEOHETERODONTA	UNIONIDA	UNIONIDAE	*ANODONTA* Lamarck, 1799			+	+				
PTERIOMORPHIA	OSTREOIDA	ANOMIIDAE	*ANOMIA* Linnaeus, 1758	+	+	+	+				
PTERIOMORPHIA	OSTREOIDA	PECTINIDAE	*ARGOPECTEN* Monterosato, 1899	+	+	+	+	+			
HETERODONTA	CHAMIDA	ASTARTIDAE	*ASTARTE* Sowerby, 1816	+	+	+	+				
HETERODONTA	VENEROIDA	BABINKIDAE	*BABINKA* Barrande, 1881							+	
PTERIOMORPHIA	ARCIDA	ARCIDAE	*BARBATIA* Gray, 1840	+	+	+	+				
HETERODONTA	VENEROIDA	BERNARDINIDAE	*BERNARDINA* Dall, 1910							+	
HETERODONTA	VENEROIDA	FIMBRIIDAE	*BERNAYIA* Cossmann, 1887							+	
PTERIOMORPHIA	OSTREOIDA	OSTREIDAE	*BOSOSTREA* Chiplonkar & Badve, 1978					+			
PALAEOHETERODONTA	MODIOMORPHOIDA	MODIOMORPHIDAE	*BYSSODESMA* Isberg, 1934					+			
HETERODONTA	VENEROIDA	CARDITIDAE	*CARDITA* Bruguière, 1792		+	+	+				
HETERODONTA	CHAMIDA	CARDIIDAE	*CARDIUM* Linne, 1758	+	+	+	+	+			
HETERODONTA	CHAMIDA	CARDIIDAE	*CERASTODERMA* Poli, 1795	+	+	+					
HETERODONTA	CHAMIDA	VENERIDAE	*CHAMELEA* Mörch, 1853								+
HETERODONTA	VENEROIDA	CHLAMYDOCONCHIDAE	*CHLAMYDOCONCHA* Dall, 1884							+	
PTERIOMORPHIA	OSTREOIDA	PECTINIDAE	*CHLAMYS* Röding, 1798	+	+	+	+				
HETERODONTA	CHAMIDA	CORBICULIDAE	*CORBICULA* Megerle von Mühlfeld, 1811	+	+						
PTERIOMORPHIA	OSTREOIDA	OSTREIDAE	*CRASSOSTREA* Sacco, 1897	+	+	+	+				
ANOMALODESMATA	PHOLADOMYOIDA	CUSPIDARIIDAE	*CUSPIDARIA* Nardo, 1840			+	+	+			
HETERODONTA	VENEROIDA	CYRENOIDIDAE	*CYRENOIDA* de Joannis, 1835							+	

Subclass	Order	Family	Genus	R1	R2	R3	R4	S1	S2	S3	S4
HETERODONTA	CHAMIDA	DONACIDAE	*DONAX* Linnaeus, 1758	+	+	+	+				+
HETERODONTA	CHAMIDA	DREISSENIDAE	*DREISSENA* Beneden, 1835	+	+	+	+				
HETERODONTA	CHAMIDA	PHARIDAE	*ENSIS* Schumacher, 1817	+	+	+	+	+			+
HETERODONTA	CHAMIDA	RZEHAKIIDAE	*ERGENICA* Zhizchenko, 1953							+	
HETERODONTA	CHAMIDA	VENERIDAE	*GAFRARIUM* Röding, 1798		+	+	+				
HETERODONTA	CHAMIDA	VENERIDAE	*GEMMA* Deshayes, 1853	+		+	+				
HETERODONTA	MYIDA	HIATELLIDAE	*HIATELLA* Daudin in Bosc, 1801	+	+	+	+				
PALAEOHETERODONTA	MODIOMORPHOIDA	MODIOMORPHIDAE	*HIPPOMYA* Salter, 1864						+		
PTERIOMORPHIA	OSTREOIDA	GRYPHAEIDAE	*HYOTISSA* Stenzel, 1971		+	+	+				
PALAEOHETERODONTA	UNIONIDA	UNIONIDAE	*HYRIOPSIS* Conrad, 1853			+	+				
PTERIOMORPHIA	MYTILIDA	MYTILIDAE	*IDAS* Jeffreys, 1876						+		
PTERIOMORPHIA	PTERIIDA	INOCERAMIDAE	*INOCERAMUS* J. Sowerby, 1814					+			
PALAEOHETERODONTA	UNIONIDA	UNIONIDAE	*INVERSIDENS* Haas, 1911	+	+	+	+				
PROTOBRANCHIA	NUCULOIDA	ISOARCIDAE	*ISOARCA* Münster, 1842						+		
PALAEOHETERODONTA	UNIONIDA	UNIONIDAE	*LAMPSILIS* Rafinesque, 1820	+	+	+	+				
HETERODONTA	VENEROIDA	LASAEIDAE	*LASAEA* Leach in Brown, 1827							+	
ANOMALODESMATA	PHOLADOMYOIDA	LATERNULIDAE	*LATERNULA* Röding, 1798						+		
PTERIOMORPHIA	LIMIDA	LIMIDAE	*LIMA* Bruguière, 1797	+	+	+	+				
PROTOBRANCHIA	NUCULOIDA	NUCULANIDAE	*LONGINUCULANA* Saveliev, 1958						+		
PTERIOMORPHIA	OSTREOIDA	OSTREIDAE	*LOPHA* Röding, 1798	+							
ANOMALODESMATA	PHOLADOMYOIDA	LYONSIIDAE	*LYONSIA* Turton, 1822					+	+		
HETERODONTA	CHAMIDA	TELLINIDAE	*MACOMA* Leach, 1819								+
HETERODONTA	CHAMIDA	MACTRIDAE	*MACTRA* Linne, 1767			+	+	+			+
ANOMALODESMATA	PHOLADOMYOIDA	MARGARITARIIDAE	*MARGARITARIA* Conrad, 1849						+		
PALAEOHETERODONTA	UNIONIDA	MARGARITIFERIDAE	*MARGARITIFERA* Schumacher, 1816					+	+		
ANOMALODESMATA	PHOLADOMYOIDA	MEGADESMIDAE	*MEGADESMUS* J. De Sowerby, 1838						+		
HETERODONTA	CHAMIDA	VENERIDAE	*MERCENARIA* Schumacher, 1817	+							
PTERIOMORPHIA	OSTREOIDA	PECTINIDAE	*MIMACHLAMYS* Iredale, 1929			+	+				+

Subclass	Order	Family	Genus	R1	R2	R3	R4	S1	S2	S3	S4
PTERIOMORPHIA	OSTREOIDA	PECTINIDAE	*MIZUHOPECTEN* Masuda, 1963	+	+	+	+				
HETERODONTA	MYIDA	MYIDAE	*MYA* Linnaeus, 1758	+	+	+	+				
PALAEOHETERODONTA	UNIONIDA	MYCETOPODIDAE	*MYCETOPODA* d'Orbigny, 1835						+		
PALAEOHETERODONTA	TRIGONIOIDA	MYOPHORIIDAE	*MYOPHORIA* Bronn, 1834						+		
PTERIOMORPHIA	MYTILIDA	MYTILIDAE	*MYTILUS* Linnaeus, 1758	+	+	+	+	+			+
PALAEOHETERODONTA	TRIGONIOIDA	NAKAMURANAIIDAE	*NAKAMURANAIA* Suzuki, 1943						+		
PROTOBRANCHIA	NUCULOIDA	NUCULIDAE	*NUCULA* Lamarck, 1799	+	+	+	+	+	+		
PROTOBRANCHIA	NUCULOIDA	NUCULANIDAE	*NUCULANA* Link, 1807			+	+	+			
PROTOBRANCHIA	PRAECARDIOIDA	CARDIOLIDAE	*ONTARIA* Clarke, 1904						+		
PTERIOMORPHIA	OSTREOIDA	OSTREIDAE	*OSTREA* Linnaeus, 1758								+
PROTOBRANCHIA	NUCULOIDA	NUCULIDAE	*PALAEONUCULA* Quenstedt, 1930						+		
ANOMALODESMATA	PHOLADOMYOIDA	PANDORIDAE	*PANDORA* Bruguière, 1797			+	+		+		
PROTOBRANCHIA	PRAECARDIOIDA	PRAECARDIIDAE	*PARACARDIUM* Barrande, 1881							+	
PTERIOMORPHIA	OSTREOIDA	PECTINIDAE	*PECTEN* Müller, 1776	+	+	+	+				+
HETERODONTA	CHAMIDA	PHARIDAE	*PHARUS* Gray, 1840								+
ANOMALODESMATA	PHOLADOMYOIDA	PHOLADOMYIDAE	*PHOLADOMYA* G. B. Sowerby I, 1823					+			
PTERIOMORPHIA	PTERIIDA	PTERIIDAE	*PINCTADA* Röding, 1798	+		+	+				
PTERIOMORPHIA	PTERIIDA	PINNIDAE	*PINNA* Linnaeus, 1758	+	+	+	+				+
PTERIOMORPHIA	OSTREOIDA	PECTINIDAE	*PLACOPECTEN* Verrill, 1897	+	+	+	+				
PALAEOHETERODONTA	UNIONIDA	UNIONIDAE	*POPENAIAS* Frierson, 1927							+	
PTERIOMORPHIA	PTERIIDA	PTERIIDAE	*PTERIA* Scopoli, 1777								+
HETERODONTA	CHAMIDA	QUENSTEDTIIDAE	*QUENSTEDTIA* Morris & Lycett, 1854							+	
HETERODONTA	CHAMIDA	RZEHAKIIDAE	*RZEHAKIA* Korobkov, 1954							+	
HETERODONTA	CHAMIDA	SCROBICULARIIDAE	*SCROBICULARIA* Schumacher, 1815							+	
PTERIOMORPHIA	PTERIIDA	MYALINIDAE	*SEPTIMYALINA* Newell, 1942							+	
HETERODONTA	CHAMIDA	PSAMMOBIIDAE	*SINONOVACULA* Prashad, 1924			+	+				
PROTOBRANCHIA	SOLEMYIDA	SOLEMYIDAE	*SOLEMYA* Lamarck, 1818		+	+	+	+			
HETERODONTA	CHAMIDA	SOLENIDAE	*SOLEN* Linnaeus, 1758								+

Subclass	Order	Family	Genus	R1	R2	R3	R4	S1	S2	S3	S4
HETERODONTA	CHAMIDA	MACTRIDAE	*SPISULA* Gray, 1837	+	+	+	+				
PTERIOMORPHIA	OSTREOIDA	SPONDYLIDAE	*SPONDYLUS* Linnaeus, 1758	+	+	+	+				
HETERODONTA	VENEROIDA	BERNARDINIDAE	*STOHLERIA* Coen, 1984							+	
HETERODONTA	CHAMIDA	TELLINIDAE	*TELLINA* Linnaeus, 1758								+
HETERODONTA	MYIDA	TEREDINIDAE	*TEREDO* Linnaeus, 1758								+
ANOMALODESMATA	PHOLADOMYOIDA	THRACIIDAE	*THRACIA* Leach in de Blainville, 1824		+	+	+	+			
HETERODONTA	CHAMIDA	VENERIDAE	*TIVELA* Link, 1807						+		
HETERODONTA	CHAMIDA	TRIDACNIDAE	*TRIDACNA* Bruguière, 1797		+	+	+				
PALAEOHETERODONTA	TRIGONIOIDA	TRIGONIIDAE	*TRIGONIA* Bruguière, 1798					+	+		
PROTOBRANCHIA	NUCULOIDA	PRAENUCULIDAE	*TRIGONOCONCHA* Sanchez, 1999						+		
PALAEOHETERODONTA	UNIONIDA	UNIONIDAE	*UNIO* Philipsson, 1788					+			
HETERODONTA	CHAMIDA	LYMNOCARDIIDAE	*UNIOCARDIUM* Capellini, 1880							+	
HETERODONTA	MYIDA	TEREDINIDAE	*UPEROTUS* Guettard, 1770							+	
HETERODONTA	CHAMIDA	VENERIDAE	*VENERUPIS* Lamarck, 1818	+	+	+	+				+
HETERODONTA	CHAMIDA	VENERIDAE	*VENUS* Linnaeus, 1758					+			+
ANOMALODESMATA	PHOLADOMYOIDA	VERTICORDIIDAE	*VERTICORDIA* Gray, 1840						+		
HETERODONTA	MYIDA	XYLOPHAGIDAE	*XYLOPHAGA* Turton, 1822							+	
PROTOBRANCHIA	NUCULOIDA	YOLDIIDAE	*YOLDIA* Möller, 1842					+			
PROTOBRANCHIA	NUCULOIDA	YOLDIIDAE	*YOLDIELLA* Verrill & Bush, 1897						+		
			Totals	31	33	42	41	20	21	20	18
